# The correlation between lipoprotein(a) elevations and the risk of recurrent cardiovascular events in CAD patients with different LDL-C levels

**DOI:** 10.1186/s12872-022-02618-5

**Published:** 2022-04-15

**Authors:** Lijun Zhu, Jiamin Zheng, Beibei Gao, Xiangbo Jin, Ying He, Liang Zhou, Jinyu Huang

**Affiliations:** 1 Zhejiang Chinese Medical University, Zhejiang, China; 2 Department of Cardiology, Hangzhou First People’s Hospital Affiliated to Zhejiang University School of Medicine, Zhejiang, China; 3 Zhejiang University School of Medicine, Zhejiang, China

**Keywords:** Lipoprotein(a) elevations, Low-density lipoprotein cholesterol, Recurrent cardiovascular events

## Abstract

**Background:**

Lipoprotein(a) [Lp(a)] elevation is an important risk factor for coronary artery disease (CAD). However, the correlation between Lp(a) elevations and the risk of recurrent cardiovascular events in patients with established cardiovascular disease is controversial. Some studies have shown that Low-density lipoprotein cholesterol (LDL-C) levels may influence the association between Lp(a) and cardiovascular risk. Our study aims to explore the correlation between Lp(a) elevations and cardiovascular risk in patients with different LDL-C levels.

**Methods:**

We included 516 patients who received coronary stents due to acute coronary syndrome (ACS) and followed them for three years. They were divided into low-Lp(a) group and high-Lp(a) group according to Lp(a) levels, and the incidence of major adverse cardiovascular events (MACE) and acute coronary events (ACE) was compared between the two groups. Then the patients were divided into three subgroups (S1:LDL-C ≥ 1.8 mmol/L; S2:1.4 ≤ LDL-C < 1.8 mmol/L; S3:LDL-C < 1.4 mmol/L). The correlation between Lp(a) elevations and cardiovascular risk in different subgroups was analysed by Cox proportional hazards models.

**Results:**

The incidence of MACE and ACE in the high-Lp(a) group was significantly higher than those in the low-Lp(a) group (*P* < 0.05). Lp(a) elevations had independent prognostic value from the statistical point of view (MACE: HR = 1.63, 95%CI = 1.12–2.38, *P* = 0.012; ACE: HR = 1.70, 95%CI = 1.03–2.81, *P* = 0.037). Subgroup analysis showed that Lp(a) elevations increased cardiovascular risk when LDL-C ≥ 1.4 mmol/L. However, this correlation no longer existed when LDL-C levels were very low (< 1.4 mmol/L) (MACE: HR = 0.49, 95%CI = 0.17–1.42, *P* = 0.186; ACE: HR = 0.68, 95%CI = 0.18–2.61, *P* = 0.570).

**Conclusions:**

Lp(a) elevations are associated with recurrent cardiovascular events when LDL-C levels are high, but this association may change when LDL-C levels are extremely low. CAD patients with combination of LDL-C ≥ 1.4 mmol/L and Lp(a) elevations shall be considered as high-risk groups and require further medication for the reduction of their LDL-C levels.

**Supplementary Information:**

The online version contains supplementary material available at 10.1186/s12872-022-02618-5.

## Introduction

CAD has a high mortality and disability rate and is one of the primary diseases threatening human health worldwide. Survivors of ACE are still at an increased risk of recurrence despite receiving standard revascularization and secondary prophylaxis treatment. It is estimated that ischemic events’ recurrence rate within one year has reached up to 9.2% after ACS [1]. Accurately identifying the risk of recurrent cardiovascular events in patients with CAD is of great significance to guide secondary prevention and improve prognosis.

Lp(a) is an isomeric protein composed of apolipoprotein(a), apolipoprotein B-100 and lipids such as cholesterol, phospholipids and triglycerides [2]. Previous studies have demonstrated that Lp(a) can promote atherosclerosis, inflammation and thrombosis, and is an independent risk factor for CAD [3, 4]. The 2019 ESC/EAS guideline for dyslipidemia management recommended that every adult should have at least one Lp(a) level assessment in their lifetime [5]. However, the value of Lp(a) in predicting recurrent cardiovascular events in CAD patients receiving secondary prevention strategies is controversial. Some studies have shown that Lp(a) elevations are associated with an increased risk of cardiovascular events in patients irrespective of LDL-C [6–8], but others have demonstrated Lp(a) is associated with plaque volume and MACE when LDL-C levels are high, and this association does not exist when LDL-C levels are low [9–11]. This study aims to explore the association between Lp(a) elevations and cardiovascular risk in patients with different LDL-C levels, especially those with very low LDL-C (< 1.4 mmol/L).

## Methods

### The studied population

In this retrospective study, we reviewed a total of 618 patients with ACS [unstable angina (UA), non-ST-elevation myocardial infarction (NSTEMI), and ST-elevation myocardial infarction (STEMI)] who received coronary stent implantation in Hangzhou First People's Hospital Affiliated to Zhejiang University School of Medicine from January 1 to December 31, 2017. All patients received standard medication recommended by national guidelines after stent implantation [12, 13]. Patients received dual antiplatelet therapy (100 mg aspirin and 75 mg clopidogrel/180 mg ticagrelor) for at least 12 months and Single antiplatelet therapy (100 mg aspirin or 75 mg clopidogrel) for lifelong. All patients received 10 mg rosuvastatin or 20 mg atorvastatin for lipid-lowering treatment (LLT), with ezetimibe added directly for baseline LDL-C greater than 3.4 mmol/L. Other drugs were prescribed at the discretion of the patient's treating physician.

Exclusion criteria: 1. The residual stenosis of target vessel after the operation was more than 20%, TIMI blood flow was less than Grade-III, or serious complications occurred; 2. The patient discontinued or changed the medication without authorization during the follow-up; 3. The LDL-C level fluctuated significantly during the follow-up; 4. Basic information was missing or blood lipids were not monitored as required; 5. the patients had the coronary artery bypass grafting history; 6. The patients had severe hepatic and renal function deficiency; 7. The patients with an estimated survival time of less than 3 years; 8. Loss of follow-up.

**Fig. 1:**
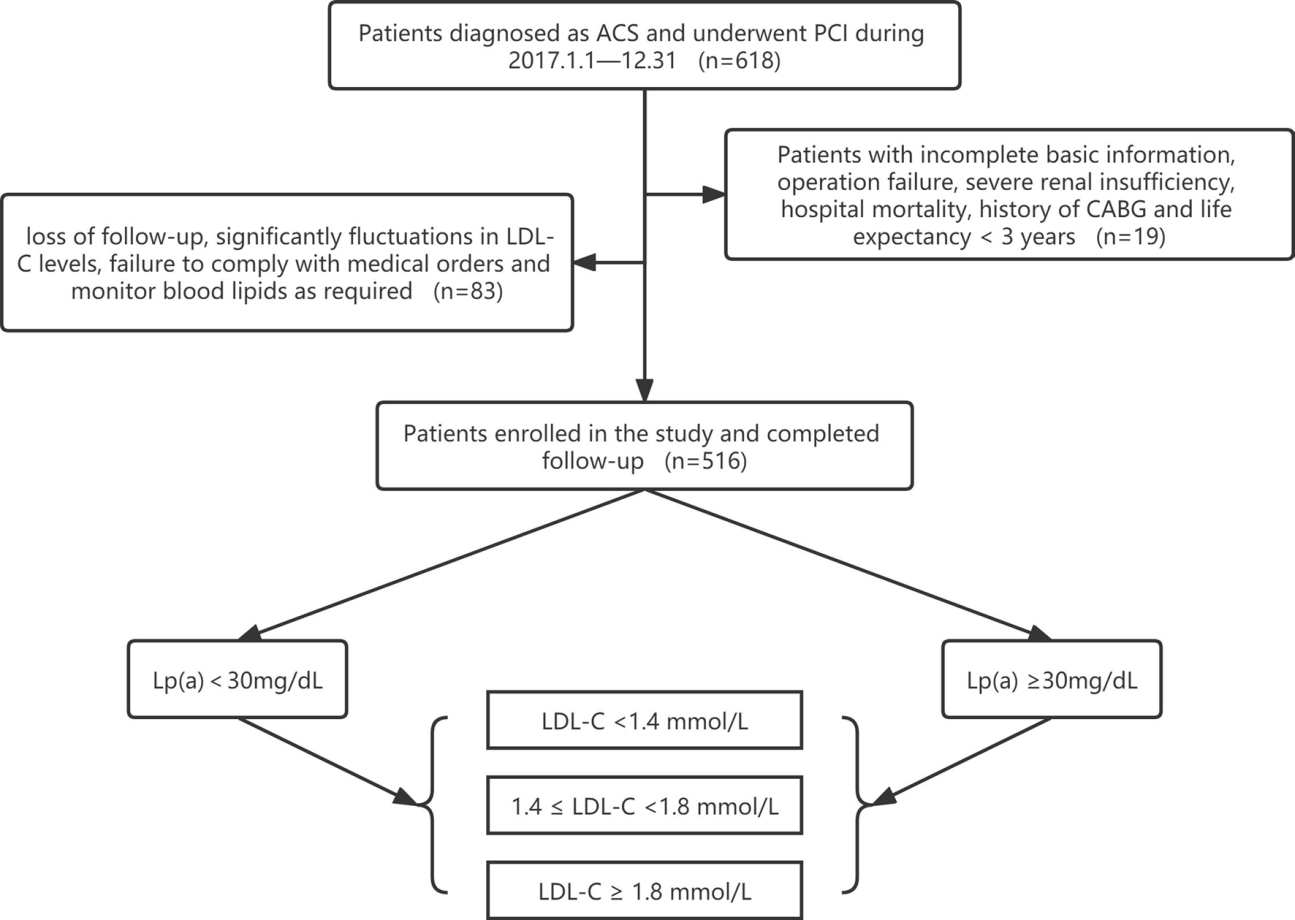
Study flow chart

Figure 1 shows the flow chart of the study. A total of 516 cases were included in this study. After 3 years of follow-up (average 31.5 months), 114 (22.1%) patients developed MACE and 64 (12.4%) patients developed ACE. It has been demonstrated that the association between Lp(a) and CAD risk is continuous and curvilinear, when Lp(a) exceeds a certain level, CAD risk increases significantly [14]. In China, this critical value is generally considered to be 30 mg/dL [15]. Therefore, we divided patients into low-Lp(a) group (< 30 mg/dL) and high-Lp(a) group (≥ 30 mg/dL) according to the Lp(a) levels at 1 month follow-up. In the subgroup analysis, they were further divided into S1 (LDL-C ≥ 1.8 mmol/L), S2 (1.4 ≤ LDL-C < 1.8 mmol/L), and S3(LDL-C < 1.4 mmol/L) according to LDL-C levels at 1 month follow-up.

### Data collection and follow-up

We recorded demographic data, cardiovascular risk factors, laboratory data and coronary angiography reports of all patients by the electronic medical record system. Cardiovascular risk factors included age, gender, body mass index, smoking status, diabetes, hypertension, and family history. All the patients received fasting blood collection before percutaneous coronary intervention (PCI), and the blood samples were measured and analyzed by the biochemical laboratory of Hangzhou First People's Hospital Affiliated to Zhejiang University School of Medicine. Coronary angiography and PCI are completed by experienced cardiovascular experts.

Patients were followed up through outpatient and inpatient records and telephone contacts for medication use, blood lipids control, and endpoint events at the 36 months after PCI. All patients received outpatient follow-up and repeated measurement of lipids in the first month after discharge. During the follow-up period after discharge, each patient had at least two blood lipids measurements.

### Study endpoints

The end points were MACE and ACE. MACE included cardiogenic death, non-fatal myocardial infarction or ischemic stroke, unplanned coronary revascularization, and hospitalization related to UA. Unplanned coronary revascularization referred to ischemia driven revascularization and stenosis driven revascularization. Stenosis driven revascularization was defined as revascularization performed when angiographic review showed significant exacerbation of stenosis without angina symptoms. ACE included STEMI, NSTEMI and UA.

### Statistical analysis

The categorical variables were represented as total cases [cases (%)] and analyzed by chi-square test or Fisher's exact test. The continuous variables that met the normal distribution were represented as mean ± SD and analyzed by t-test or variance analysis. Continuous variables with non normal distribution were represented as median (interquartile interval) and analyzed by nonparametric test. Kaplan–Meier curve and the log-rank test were performed to compare the event-free survival among the groups. Cox proportional hazards models were performed to determine the hazard ratio (HR) and 95% confidence interval (CI) for MACE and ACE. All the reported p values in this study are 2-sided and *P* < 0.05 indicated statistical significance.

## Results

### Baseline characteristics

The average age of the patients involved in this study was 66.0 ± 9.8 years, including 350 males (67.8%). As shown in Table 1, the levels of total cholesterol (TC), LDL-C and triglyceride were significantly decreased after LLT and lifestyle improvement (*P* < 0.05). The Lp(a) levels at 1-month follow-up were higher than the baseline (*P* < 0.05), which related to the use of statins. And there was no notable change in the high-density lipoprotein cholesterol (HDL-C) levels (*P* > 0.05). Table 2 summarized the baseline characteristics of the patients.

**Table 1:** Blood lipids levels at baseline and 1-month follow-up

Variables	Baseline	1-Month follow-up	*P*	Percent change
TC (mmol/L)	4.34 ± 1.16	3.30 ± 0.70	< 0.001	− 21.34%
LDL-C (mmol/L)	2.50 ± 0.94	1.64 ± 0.54	< 0.001	− 29.88%
HDL-C (mmol/L)	1.08 ± 0.27	1.09 ± 0.87	0.815	4.88%
Lipoprotein(a) (mg/dL)	13.0 (8.0–26.75)	15 (8.0–35.0)	0.040	19.27%
Triglyceride (mmol/L)	1.65 ± 1.25	1.35 ± 0.76	< 0.001	− 8.07%

**Table 2:** Characteristics of the study population

Variables	Low-Lp(a) (n = 365)	High-Lp(a) (n = 151)	*P*
Age [years]	65.60 ± 10.30	67.10 ± 8.46	0.115
Male [cases (%)]	259 (70.96)	91 (60.26)	0.018
BMI (kg/m^2^)	24.29 ± 3.00	24.24 ± 3.38	0.878
Systolic pressure (mmHg)	126.13 ± 13.73	128.38 ± 15.18	0.116
Diastolic pressure (mmHg)	68.92 ± 10.16	69.03 ± 9.69	0.911
Former smokers [cases (%)]	175 (47.95)	63 (41.72)	0.197
Current smokers [cases (%)]	26 (7.12)	8 (5.30)	0.447
Hypertension history [cases (%)]	270 (73.97)	120 (79.47)	0.186
Diabetes history [cases (%)]	119 (32.60)	55 (36.42)	0.404
Family history [cases (%)]	32 (8.77)	10 (6.62)	0.418
Creatinine (μmoI/L)	86.99 ± 16.71	90.77 ± 22.01	0.034
hs-CRP (mg/L)	3.00 (1.00–5.00)	3 (1.00–6.00)	0.252
Single-vessel lesions [cases (%)]*	111 (30.41)	39 (25.83)	0.243
Double-vessel lesions [cases (%)]	112 (30.68)	39 (25.83)	0.219
Triple-vessel lesions [cases (%)]	142 (38.90)	73 (48.34)	0.048
Left main lesions [cases (%)]	36 (9.86)	15 (9.93)	0.980
Total coronary occlusion [cases (%)]	63 (17.26)	40 (26.49)	0.017
Coronary stents (number)	2.41 ± 1.59	2.64 ± 1.61	0.132
*Baseline lipids levels*			
TC (mmol/L)	4.24 ± 1.18	4.60 ± 1.08	0.001
LDL-C (mmol/L)	2.43 ± 0.97	2.68 ± 0.84	0.003
HDL-C (mmol/L)	1.05 ± 0.27	1.14 ± 0.29	0.001
Lp(a) (mg/dL)	10.0 (7.0–14.0)	42.0 (28.0–74.0)	< 0.001
Triglyceride (mmol/L)	1.62 ± 1.01	1.71 ± 1.69	0.569
*Lipids levels at 1-month follow-up*			
TC (mmol/L)	3.23 ± 0.71	3.48 ± 0.65	< 0.001
LDL-C (mmol/L)	1.60 ± 0.55	1.75 ± 0.50	0.002
HDL-C (mmol/L)	1.08 ± 1.01	1.11 ± 0.28	0.606
Lp(a) (mg/dL)	11.0 (7.0–16.0)	52.6 (37.0–80.0)	< 0.001
Triglyceride (mmol/L)	1.36 ± 0.79	1.33 ± 0.71	0.654
*Medication use after discharge*			
Statins [cases (%)]	365 (100)	151 (100)	1
Ezetimibe [cases (%)]	27 (7.40)	7 (4.64)	0.25
Aspirin [cases (%)]	365 (100)	151 (100)	1
Clopidogrel [cases (%)]	285 (78.08)	118 (78.15)	0.987
Ticagrelor [cases (%)]	80 (21.92)	33 (21.85)	0.987
*Medication use before the endpoints*			
Statins [cases (%)]	365 (100)	151 (100)	1
Ezetimibe [cases (%)]	22 (6.03)	7 (4.64)	0.532
Dual antiplatelet therapy [cases (%)]	30 (8.22)	19 (12.58)	0.124
Aspirin [cases (%)]	30 (8.22)	19 (12.58)	1
Clopidogrel [cases (%)]	21 (5.75)	15 (9.93)	0.489
Ticagrelor [cases (%)]	9 (2.47)	4 (2.65)	0.489
Single antiplatelet therapy [cases (%)]	335 (91.78)	132 (87.42)	0.124
Aspirin [cases (%)]	296 (81.10)	119 (78.81)	0.579
Clopidogrel [cases (%)]	39 (10.68)	13 (8.61)	0.579
Ticagrelor [cases (%)]	0	0	–

^*^Lesions were defined as coronary atherosclerotic stenosis of more than 50%

The baseline TC (4.60 ± 1.08 vs. 4.24 ± 1.18, *P* = 0.001), LDL-C (2.68 ± 0.84 vs. 2.43 ± 0.97, *P* = 0.001) and HDL-C (1.14 ± 0.29 vs. 1.05 ± 0.27, *P* = 0.001) in the high-Lp(a) group were higher than those in the low-Lp(a) group. At 1-month follow-up, the TC (3.48 ± 0.65 vs. 3.23 ± 0.71, *P* < 0.001) and LDL-C (1.75 ± 0.50 vs. 1.60 ± 0.55, *P* = 0.002) in the high-Lp(a) group were still higher, but there was no statistical difference in HDL-C between the two groups (1.11 ± 0.28 vs. 1.08 ± 1.01, *P* = 0.606). Higher creatinine (90.77 ± 22.01 vs. 86.99 ± 16.71, *P* = 0.034), female (39.74% vs. 29.04%, P = 0.018), total coronary occlusion (26.49% vs. 17.26%, *P* = 0.017) and Triple-vessel lesion (48.34% vs. 38.90%, *P* = 0.048) were more common to be found in the high-Lp(a) group. There was no statistical significance in other characteristics between the two groups (*P* > 0.05).

### Endpoint events

After 3 years of follow-up (average 31.5 months), 114 MACE and 64 ACE were recorded. As shown in Table 3, The incidence of MACE (*P* = 0.003) and ACE (*P* = 0.006) in the High-Lp(a) group was significantly higher than that in the low-Lp(a) group. Further analysis of MACE showed that the rates of hospitalization related to UA (*P* = 0.026) and unplanned coronary revascularization (*P* = 0.008) were higher in high-Lp(a) group. The overall incidence of cardiogenic death and nonfatal myocardial infarction or ischemic stroke was low, and there was no statistical difference observed between the two groups (*P* > 0.05).

**Table 3:** Comparison of endpoint events incidence between high-Lp(a) and low-Lp(a) group

Endpoint events	Low-Lp(a) (n = 365)	High-Lp(a) (n = 151)	*P*
Major adverse cardiovascular events	68 (18.63)	46 (30.46)	0.003
Cardiogenic death	7 (1.92)	4 (2.65)	0.633
Non-fatal MI or ischemic stroke	15 (4.11)	10 (6.62)	0.226
Hospitalization related to UA	26 (7.12)	20 (13.25)	0.026
Unplanned coronary revascularization	46 (12.60)	33 (21.85)	0.008
Acute coronary events*	36 (9.86)	28 (18.54)	0.006

^*^Acute coronary events included STEMI, NSTEMI and UA

**Fig. 2:**
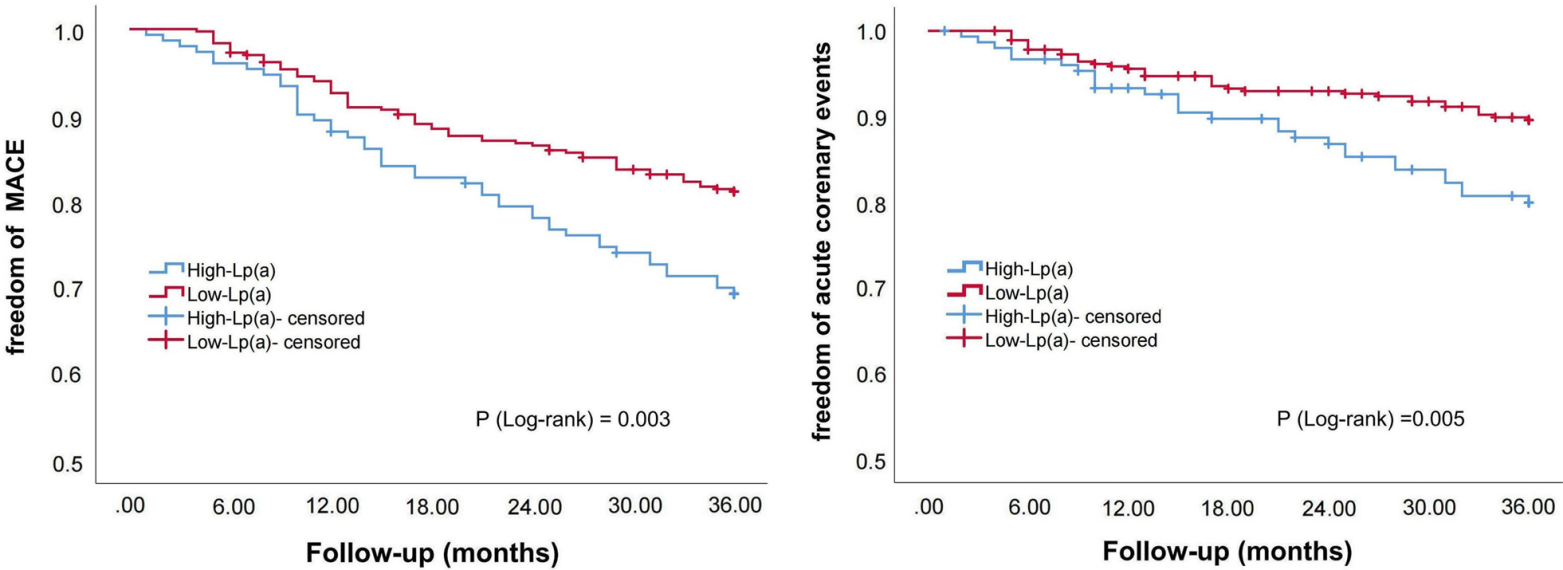
The Kaplan–Meier survival curves analysis of the entire sample

**Fig. 3:**
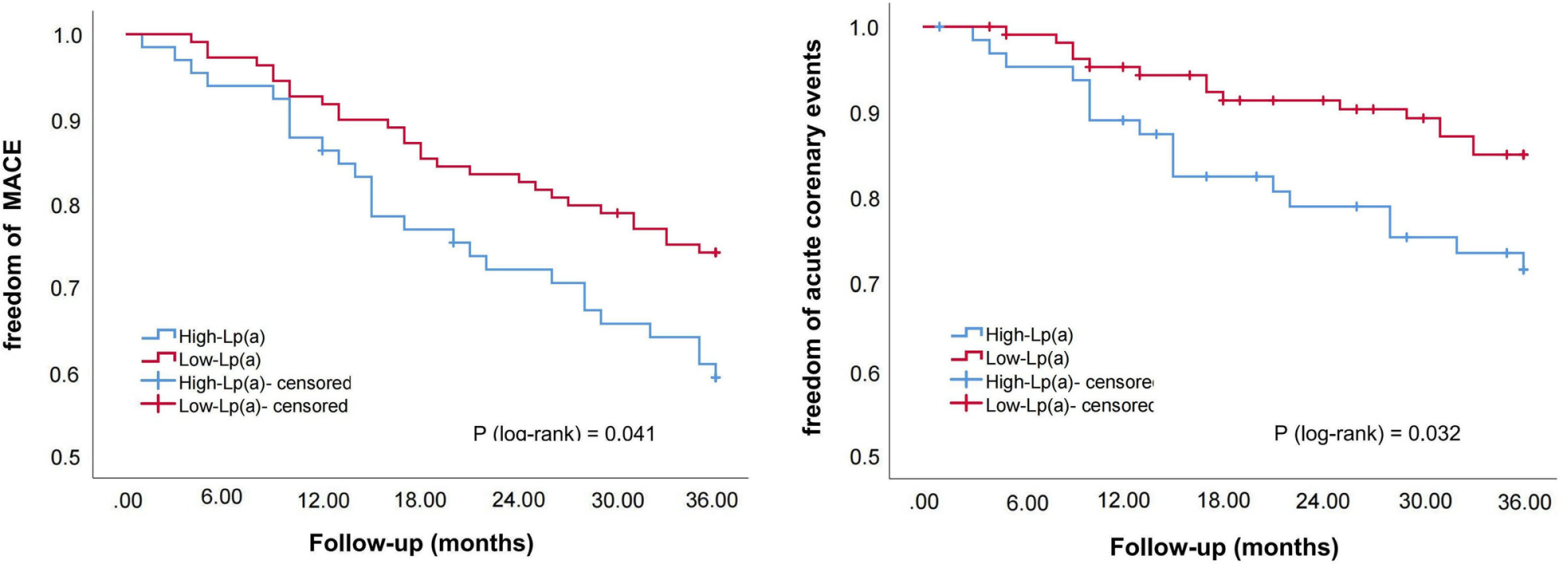
The Kaplan–Meier survival curves analysis of S1

**Fig. 4:**
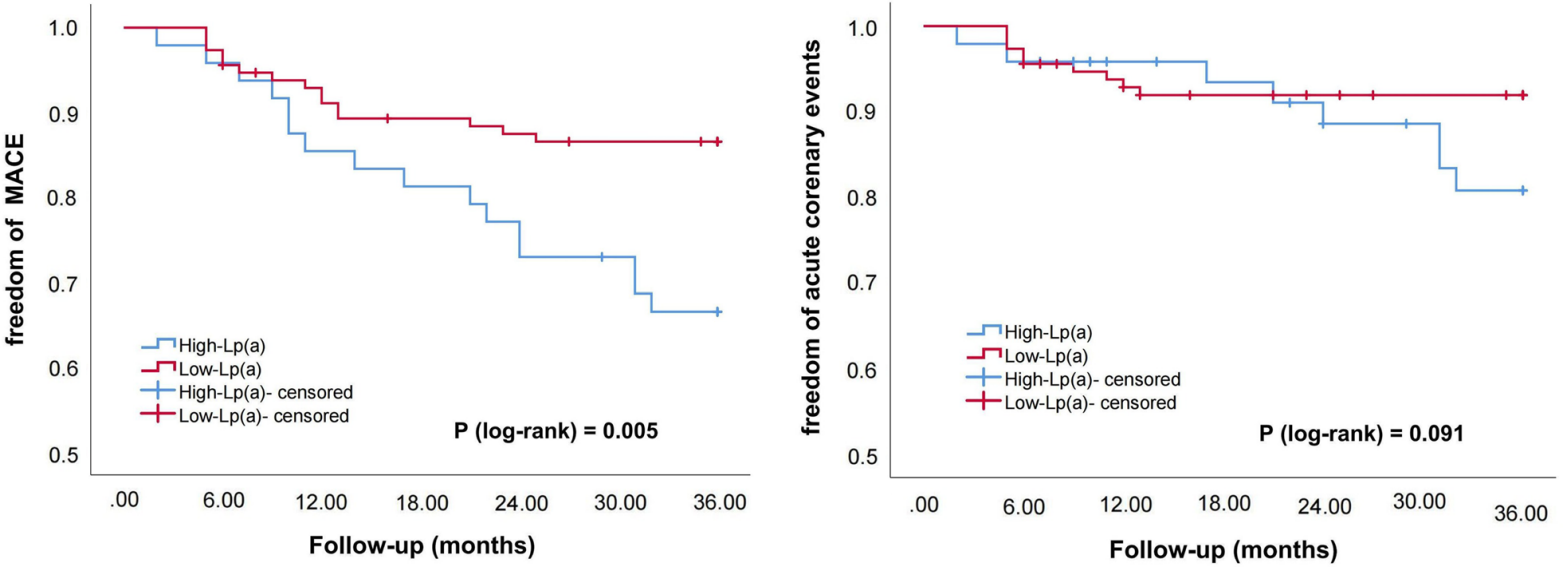
The Kaplan–Meier survival curves analysis of S2

**Fig. 5:**
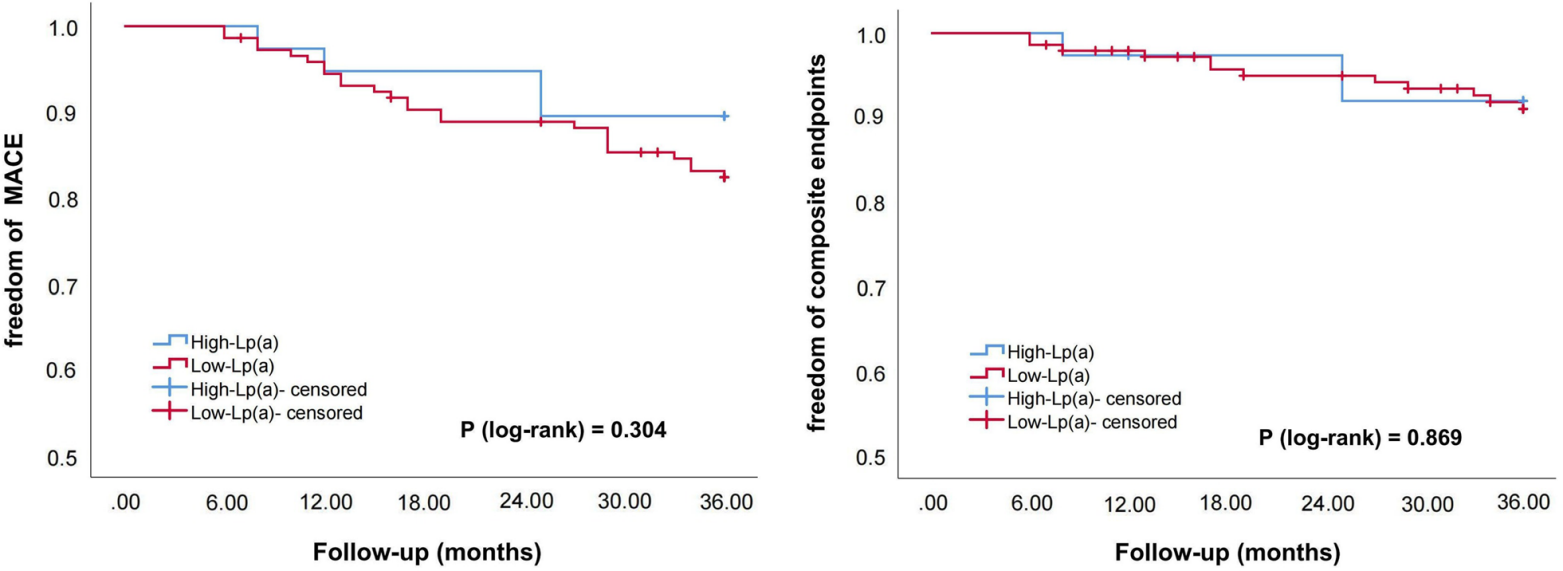
The Kaplan–Meier survival curves analysis of S3

Subsequently, we divided the patients into three subgroups according to LDL-C levels (S1: LDL-C ≥ 1.8 mmol/L; S2: 1.4 ≤ LDL-C < 1.8 mmol/L; S3: LDL-C < 1.4 mmol/L; S0: the entire sample). Kaplan–Meier survival curve was used to compare the correlation between Lp(a) elevations and cardiovascular outcomes in the entire sample and subgroups. As shown in Figs. 2, 3, 4 and 5, Lp(a) elevations were associated with a higher incidence of MACE and ACE in S0 and S1 (*P* < 0.05). For patients with intermediate levels of LDL-C (S2), Lp(a) elevations were closely related to MACE (*P* < 0.05). The incidence of ACE in the high-Lp (a) group was also higher than that in the low-Lp (a) group, but the difference was not statistically significant (*P* > 0.05). Lp(a) elevations in S3 had no correlation with MACE and ACE, even showed an opposite trend.

Cox regression analysis was performed to determine the HR and 95% CI for MACE and ACE. As shown in Table 4, after adjusting for confounding factors such as LDL-C, elevated Lp(a) remained an important risk factor for MACE (HR = 1.63, 95%CI = 1.12–2.38, *P* = 0.012) and ACE (HR = 1.70, 95%CI = 1.03–2.81, *P* = 0.037). However, in subgroup analysis, we found that this association was not maintained in patients with LDL-C < 1.4 mmol/L. Patients with LDL-C ≥ 1.8 mmol/L and high-Lp(a) had a 2.33-fold increased risk of recurring ACE than those with low-Lp(a) (HR = 2.33, 95%CI = 1.15–4.72, *P* = 0.019), and the risk of MACE was also relatively higher (HR = 1.62, 95% CI = 0.95–2.77). In the subgroup of LDL-C between 1.4 and 1.8 mmol/L, patients with Lp(a) elevations had a significantly higher risk of MACE (HR = 2.65, 95%CI = 1.31–5.36, *P* = 0.007). They were also at higher risk of ACE, but the difference was not statistically significant (HR = 2.01, 95% CI = 0.77–5.23). On the contrary, neither MACE (HR = 0.49, 95% CI = 0.17–1.42, *P* = 0.186) nor ACE (HR = 0.68, 95% CI = 0.18–2.61, *P* = 0.570) had any correlation with Lp(a) elevations when LDL-C < 1.4 mmol/L.

**Table 4:** Univariate and multivariate analysis of correlation between Lp(a) elevations and endpoint events in different subgroups

Endpoint events	Group	Unadjusted		Adjusted*	
		*P*	HR (95%CI)	*P*	HR (95%CI)
MACE	S0	0.004	1.74 (1.20–2.53)	0.012	1.63 (1.12–2.38)
	S1	0.045	1.73 (1.01–2.95)	0.076	1.62 (0.95–2.77)
	S2	0.007	2.65 (1.31–5.36)	0.007	2.65 (1.31–5.36)
	S3	0.312	0.58 (0.20–1.67)	0.186	0.49 (0.17–1.42)
	S2 + S3	0.121	1.53 (0.89–2.62)	0.172	1.46 (0.85–2.51)
ACE	S0	0.006	2.00 (1.22–3.28)	0.037	1.70 (1.03–2.81)
	S1	0.036	2.10 (1.05–4.21)	0.019	2.33 (1.15–4.72)
	S2	0.101	2.22 (0.86–5.76)	0.152	2.01 (0.77–5.23)
	S3	0.869	0.90 (0.25–3.19)	0.570	0.68 (0.18–2.61)
	S2 + S3	0.203	1.61 (0.77–3.33)	0.232	1.56 (0.75–3.24)

S0: the entire sample; S1: LDL-C ≥ 1.8 mmol/L; S2: 1.4 ≤ LDL-C < 1.8 mmol/L; S3: LDL-C < 1.4 mmol/L**;** S2 + S3: LDL-C < 1.8 mmol/L

^*^The variables with *P* < 0.05 in univariate analysis were included in multivariate COX regression analysis. The results of univariate analysis are listed in Additional file 1: Table S1

## Discussion

Our study demonstrated that the correlation between Lp(a) and cardiovascular risk is affected by LDL-C levels. In patients with LDL-C ≥ 1.4 mmol/l, Lp(a) elevations are closely related to MACE and ACE, but there is no correlation in patients with LDL < 1.4 mmol/l. The results emphasize the importance of controlling LDL-C in patients with CAD once again, and provides a new reference for the formulation of lipid-lowering strategies. CAD patients with combination of LDL-C ≥ 1.4 mmol/L and Lp(a) elevations shall be considered as high-risk groups and require further medication for the reduction of their LDL-C levels.

Controlling LDL-C is the cornerstone of the prevention and treatment of atherosclerotic cardiovascular diseases (ASCVD). To further reduce cardiovascular risk, the 2019 ESC/EAS guideline suggested that the LDL-C levels in patients with extremely high risk ASCVD should be controlled below 1.4 mmol/L [5]. According to the guideline, the LDL-C control target of patients with CAD is less than 1.4 mmol/L. However, the current lipids control of CAD patients is not ideal. There are more than 126 million people worldwide suffering from CAD [16], but less than 16% of them achieve the target of LDL-C < 1.4 mmol/L [17]. The risk of recurrent cardiovascular events varies greatly among patients with CAD. Hence, it is of great significance to accurately identify patients at high risk of recurrent cardiovascular events for the guidance of intensive LLT.

Lp(a) is a low-density lipoprotein-like particle that has long been regarded as a risk factor for cardiovascular disease. Prior research has confirmed that Lp(a) elevations are independently associated with the risk of CAD [4, 18]. However, the correlation between Lp(a) elevations and recurrent cardiovascular risk in patients with CAD is controversial. Many studies have demonstrated Lp(a) elevations are not predictive for cardiovascular outcomes in patients medically well controlled [19–21]. Considering that LDL-C levels are higher in general population and lower in CAD patients receiving LLT, some scholars attribute the differences in the association between Lp(a) and cardiovascular risk across studies to differences in LDL-C levels. They claim that Lp(a) levels are associated with plaque load and MACE at the high LDL-C levels, and this association has changed when LDL-C levels decreased [9, 22]. On the contrary, some studies have shown that Lp(a) elevations are associated with an increased risk of cardiovascular events in patients with CAD irrespective of LDL-C [6, 7]. A recent study conducted a separate analysis of CAD patients with LDL-C ≤ 1.8 mmol/L and found Lp(a) levels are independently associated with MACE and recurrent MI in patients with well-controlled LDL-C [23].

We carried out this study to better understand the association between Lp(a), LDL-C and recurrent cardiovascular events in patients with CAD. According to the study results, we support the view that the correlation between Lp(a) and the risk of recurrent cardiovascular events was affected by the levels of LDL-C. The mechanism leading to this unique association is not yet clear. It has been reported that The degradation of Lp(a) is partly mediated by the LDL receptors (LDL-R), high-levels of LDL-C may occupy the receptors, competitively inhibit Lp(a) catabolism and enhance the biological effect of Lp(a) [24]. We speculate that patients with very low LDL-C levels tend to have high levels or activity of LDL-R and strong metabolism capacity for Lp(a). Even if Lp(a) levels are high, they can be timely metabolized and the biological effects are weakened. On the contrary, patients with high LDL-C have low levels or activity of LDL-R, Lp(a) can not be metabolized efficiently so that the biological effects are amplified. In order to prove that our results are not caused by accidental factors, we analyzed the patients with LDL-C ≤ 1.8 mmol/L (S2 + S3) and found that there was a positive correlation between Lp(a) elevations and endpoint events, which was consistent with the report of Ren et al. [23]. In addition, when analyzing the entire sample, we found that Lp(a) elevations were independently related to MACE and ACE, which were also consistent with prior reports [6, 7]. Since the proportion of patients with combination of extremely low LDL-C and elevated Lp(a) is very small in clinical practice, we speculate that they play a small role in the statistical analysis of the entire sample and can be easily masked. Therefore, different results were obtained in the holistic analysis and subgroup analysis.

The plasma concentration of Lp(a) tends to remain constant throughout life[25]. But the levels of Lp(a) increased after treatment in our study. It could be attributed to statins. A meta-analysis have shown that statin treatment is associated with an 11% increase in the geometric mean of Lp(a) concentrations compared to placebo[26]. Therefore, monitoring Lp(a) after treatment may be more helpful than baseline Lp(a) in predicting the cardiovascular risk of CAD patients. The recent guidelines and expert consensus emphasize that the cardiovascular risk of CAD patients should be further stratified to identify the most vulnerable patients who need to control LDL-C < 1.4 mmol/L [27–29]. Based on our results, we believe the levels of Lp(a) after treatment are helpful to identify the patients at high risk of recurrent cardiovascular events. The patients with combination of LDL-C ≥ 1.4 mmol/L and Lp(a) elevations shall be considered as the vulnerable ones and need to further reduce their LDL-C levels below 1.4 mmol/L.

## Limitations

Firstly, this is a single-center study including all ACS patients who underwent PCI in Hangzhou First People's Hospital Affiliated to Zhejiang University School of Medicine within one year. The sample size is relatively small (n = 516). In some results, HR and 95%CI were valuable, but statistically insignificant (*P* > 0.05). This may be due to the insufficient sample size, so our conclusions need to be further verified by larger clinical studies. Secondly, indicators such as LDL-C differ between the acute and stable phases of CAD. However, most patients measured blood lipids only at admission and during a specific follow-up period. Therefore, there is a lack of data to explain the effect of ACS induced lipids fluctuations on the results.

## Conclusions

Lp(a) elevations are associated with recurrent cardiovascular events when LDL-C levels are high, but this association may change when LDL-C levels are extremely low. Patients with combination of LDL-C ≥ 1.4 mmol/L and Lp(a) elevations shall be considered as high-risk groups and require further medication for the reduction of their LDL-C levels. In recent years, some new treatments have been shown to reduce both LDL-C and Lp(a) levels, such as antisense oligonucleotides and PCSK9 inhibitors, which will certainly bring more benefits to the high-risk patients with CAD.

## Supplementary Information

**Additional file 1.** Univariate COX analysis results.

## Data Availability

The datasets used and analyzed during the current study are available from the corresponding author on reasonable request.

## Abbreviations

Lp(a): Lipoprotein(a)
CAD: Coronary artery disease
ACS: Acute coronary syndrome
MACE: Major adverse cardiovascular events
ACE: Acute coronary events
UA: Unstable angina
NSTEMI: Non-ST-elevation myocardial infarction
STEMI: T-elevation myocardial infarction
LLT: Lipid-lowering therapy
TC: Total cholesterol therosclerotic
LDL-C: Low-density lipoprotein cholesterol
HDL-C: High-density lipoprotein cholesterol
ASCVD: Atherosclerotic cardiovascular disease
LDL-R: Low-density lipoprotein receptors
